# Indirect associations between adolescent ADHD and/or oppositional defiant disorder symptoms and adult incomes: the mediating roles of education and co-occurring psychiatric disorders

**DOI:** 10.1007/s00787-025-02842-2

**Published:** 2025-09-02

**Authors:** Sampo Seppä, Sanna Huikari, Marko Korhonen, Tanja Nordström, Tuula Hurtig, Anu-Helmi Halt

**Affiliations:** 1 Research Unit of Clinical Medicine, University of Oulu, Oulu, Finland; 2 Department of Economics, Accounting and Finance, University of Oulu, Oulu, Finland; 3 Northern Finland Birth Cohorts, Arctic Biobank, Infrastructure for Population Studies, Faculty of Medicine, University of Oulu, Oulu, Finland; 4 Research Unit of Population Health, University of Oulu, Oulu, Finland; 5 Clinic of Child Psychiatry, University of Oulu, Oulu, Finland; 6 Oulu University Hospital and University of Oulu, Medical Research Center, Oulu, Finland

**Keywords:** Attention-deficit/hyperactivity disorder, Oppositional defiant disorder, Education, Income

## Abstract

This longitudinal, population-based cohort study examines the direct and indirect associations between adolescent Attention-Deficit/Hyperactivity Disorder (ADHD) and/or Oppositional Defiant Disorder (ODD) symptoms and adult incomes. Moving beyond a simple direct link, our model explores how ADHD and/or ODD symptoms indirectly affect the accumulation of human, social, and health capital, which in turn may affect productivity and income. The population was drawn from members of the Northern Finland Birth Cohort 1986 (NFBC1986) who had ADHD and ODD symptoms assessed at age 16 using the Strengths and Weaknesses of ADHD Symptoms and Normal Behaviours (SWAN) scale. The results indicate a significant indirect negative effect of adolescent ADHD and ADHD + ODD symptoms on adult incomes. This effect was most pronounced in males with ADHD + ODD, who experienced a 25% income reduction via human capital (education) and an 18% reduction via health capital (presence of psychiatric disorders other than ADHD or ODD). Social capital did not mediate the association. The model was adjusted for work experience, white-collar status, marital status, parenthood, self-rated health, educational attainments of the participants’ parents and family type during adolescence. Notably, no direct effect of adolescent ADHD and/or ODD symptoms on adult incomes was observed. These findings highlight the potential of educational and healthcare investments to reduce the income disparities associated with ADHD and ODD symptoms in the general population.

**Supplementary Information:**

The online version contains supplementary material available at 10.1007/s00787-025-02842-2.

## Introduction

Longitudinal studies have indicated that symptoms of Attention-Deficit/Hyperactivity Disorder (ADHD) are associated with an increased risk of unemployment [1], and that employees with ADHD tend to perform less effectively than those without this condition [2]. Both individual and socioeconomic concerns arise due to the negative impact of ADHD on one’s ability to cope in the workplace, and income disparities between such individuals and the general population can stem from ADHD-specific symptoms, comorbid conditions, educational difficulties, or various social factors correlated with ADHD. One of the most robust predictors of the consequences in terms of adult incomes is education [3].

Oppositional Defiant Disorder (ODD) may also affect various socioeconomic indicators, such as educational level, occupational status, and income, so that some studies have found that symptoms of ODD are associated with a lower annual income than for controls [4, 5], while others have not found any such association [6, 7]. Given the somewhat limited previous research, it has been hypothesized that education may also mediate these effects [8].

Although ADHD and ODD are classified as distinct disorders, they frequently co-occur, with comorbidity rates estimated at 50–60% in population-based samples [9, 10]. Prior research has shown that individuals with comorbid ADHD and ODD experience more severe and persistent functional impairments than those with either disorder alone [11]. Accordingly, this study examines both the independent and combined effects of ADHD and ODD symptoms on adult income to assess their additive and interactive impacts. Given the well-documented sex differences in the expression, prevalence, and functional outcomes associated with both ADHD and ODD [12, 13], analyses were stratified by sex to allow for the detection of potentially divergent patterns between males and females.

Although ADHD and ODD symptoms are associated with reduced income, the factors mediating these challenges are not well understood. To move beyond a simple association between adolescent ADHD/ODD symptoms and adult income, and to gain a deeper understanding of the mechanisms underlying this relationship, we employ mediation analysis. The motivation for estimating the indirect effects stems from the idea that ADHD/ODD symptoms may influence an individual’s developmental trajectory and life-course outcomes. These effects may manifest through early-life factors such as educational attainment and social relationships, as well as later-life factors including employment stability and health-related challenges. These life-course pathways are linked to the accumulation of human, social, and health capital, which in turn are known to shape adult income [14–16].

Empirical evidence supports this conceptual framework. Adolescents with ADHD/ODD exhibit lower educational attainment [4, 17], strained interpersonal relationships and diminished trust [18, 19], and higher rates of additional psychiatric conditions [20, 21], all of which correspond to deficits in the three capital domains that underpin long-term socioeconomic well-being [14, 16, 22].

We hypothesize that the impact of adolescent ADHD/ODD symptoms on income are partially mediated through these capital constructs. Although mediation analysis is relatively straightforward statistically, it is appropriate for exploring potential mechanisms through which ADHD/ODD symptoms may affect income, i.e., through the mediating role of human, social, and health capital. The validity of this approach depends on several assumptions, most notably the assumption that ADHD and ODD symptoms are exogenous to the outcome (income) and to the mediators, conditional on observed covariates. We rely on the developmental stability and early-onset nature of ADHD and ODD, typically diagnosed in childhood and known to persist over time, as justification for treating these variables as plausibly exogenous for mediation purposes [23, 24]. Thus, mediation analysis provides a theoretically grounded and statistically appropriate method to identify potential indirect pathways through which adolescent ADHD and ODD symptoms may impact income outcomes later in life.

Understanding the long-term socioeconomic consequences of adolescent ADHD and ODD symptoms—and the pathways through which they operate—is essential for informing early intervention and public policy. By identifying the educational, social, and health-related mechanisms that mediate income disparities, our findings may inform targeted strategies to support occupational success among individuals with early-onset behavioral and neurodevelopmental conditions.

## METHODS

### Sample description

The data were obtained from the Northern Finland Birth Cohort 1986 (NFBC1986), a comprehensive, ongoing follow-up study that initially included 9,432 live-born children with expected birth dates between July 1, 1985, and June 30, 1986. The cohort represents 99% of all children born alive in Finland’s two northernmost provinces during that period [25, 26].

In the follow-up at the age of 16 years, questionnaires based on the Strengths and Weaknesses of ADHD-Symptoms and Normal-Behaviours (SWAN) scale [27] were sent to the parents of 9,215 adolescents whose addresses were known. A total of 6,985 parents (75.8%) returned the completed questionnaires along with a statement of informed consent. The Ethical Committee of the Northern Ostrobothnia Hospital District approved the study (Cohort 1986: Northern Ostrobothnia Hospital District Ethical Committee 108/2017 (15.1.2018)).

The SWAN scale, a revision of the SNAP-IV scale [27], was initially used to assess ADHD and ODD symptoms based on items directly linked to DSM-IV-TR [28] criteria. These items evaluated attention, hyperactivity/impulsivity, and disruptive behaviour on a seven-point scale, ranging from “Far Below Average” to “Far Above Average,” with “Average” as the midpoint. This approach allowed for a normally distributed assessment of behavioural traits.

### Exposure Variables: Symptoms of ADHD and ODD

ADHD symptoms identified across the Inattentive, Hyperactive-Impulsive, and Combined scales were assessed using 18 items, ranging from “Gives close attention to detail and avoids careless mistakes” to “Enters into conversations and games (controls interrupting/intruding)”. Additionally, ODD symptoms were assessed using eight items ranging from “Controls temper” to “Controls spitefulness and vindictiveness”. One missing item was allowed in each subscale and replaced with the mean of that subscale for the adolescent concerned.

Following the scale developer’s recommendation [29] and in alignment with previous research [30], the 95th percentile of the distribution was taken as the cut-off point for identifying symptoms. Participants were categorized into four groups based on their symptom profiles: (1) those with only ADHD symptoms, (2) those with only ODD symptoms, (3) those exhibiting both ADHD and ODD symptoms and (4) controls, who exhibited neither ADHD nor ODD symptoms.

The 95th percentile cutoff for ADHD and ODD symptoms was determined based on the distribution of the total sample rather than sex-specific distributions. This approach was chosen to maintain a consistent threshold across sexes, reflecting population-level clinical risk as would be applied using standardized norms in diagnostic settings. The SWAN scale has proved reliable and valid in previous studies, and thus offers a solid foundation for assessing behavioural problems in adolescents [31, 32].

### Outcome Variable: Income

The outcome variable was individual gross income in 2016, when participants were 30 years old. Participants with zero income were excluded from the analyses to avoid distortion in the log-transformation and to ensure interpretability of percentage-based estimates. Following recent methodological guidance [33], income was modelled as a continuous variable using a natural logarithmic transformation to reduce skewness and improve interpretability. This approach allows effect estimates to be expressed as percentage differences and aligns with best practices for income modelling in population-based studies. Income data were obtained from the Finnish Tax Administration (https://www.vero.fi/en).

### Mediators

This study draws on a theoretical framework combining human, social, and health capital models to examine how adolescent ADHD and/or ODD symptoms may influence adult income. To reduce model complexity and minimize multicollinearity, one proxy variable was selected a priori for each capital domain: educational attainment for human capital, interpersonal trust for social capital, and the presence of psychiatric disorders other than ADHD or ODD for health capital (Table 1). These choices were guided by both theoretical frameworks and prior empirical research linking these domains to income and labour market functioning [34, 35].

**Table 1:** Variables and their definitions

Variable name	Definition
Human capital	
Tertiary education	= 1 if the highest educational level attained by the age of 30 (in 2016) was tertiary education, including short-cycle tertiary education, a bachelor’s, master’s or doctoral degree or the equivalent level. 0 = others. Data on educational levels were retrieved from the Statistics Finland register.
Work experience	= a continuous variable ranging from 0 to 6, representing successive years in a specific employment category between 2011 and 2016. Data were obtained from the Statistics Finland Classification of Occupations and Socioeconomic Groups, as recorded annually for cohort members.
White collar status	= 1 if the subject´s socioeconomic status at age 30 (in 2016) was classified as either upper or lower white-collar. 0 = others. Data on socioeconomic status were retrieved from the corresponding Statistics Finland register.
Social capital	
Marital status	= 1 if marital status at age 30 (in 2016) was married or in a registered partnership. 0 = others. Data were retrieved from the register of the Digital and Population Data Services Agency.
Parenthood	= 1 if the subject had ever had a child through age 30 (in 2016). 0 = if the subject had never had a child. Data were retrieved from the register of the Digital and Population Data Services Agency.
Trust	= 1 if the participant mentioned at least one good friend at the age of 16 in answer to the question: “Do you have a close friend with whom you can confidentially discuss your matters?” 0 if the respondent gave a negative answer.
Health capital	
Self-rated health	= 1 if the answer to the question: “How would you describe your health at the moment?” was “very poor”, “poor”, or “moderate” at the age of 16. 0 if the answer was “good” or “very good”.
Psychiatric disorders (other than ADHD or ODD)	= 1 if yes. 0 = others. Data on psychiatric diagnoses other than ADHD or ODD were sourced from the Health Care Register maintained by the Finnish Institute for Health and Welfare.
Outcome variable:	
Annual income	Individual-level gross income data for cohort members in 2016 were obtained from the Finnish Tax Administration register (https://www.vero.fi/en).
Covariates	
Educational attainments of the participants´ parents	1 = at least one parent completed tertiary education (i.e., a university or university of applied sciences degree) and 0 for all other educational levels.
Family type during adolescence	1 = Family with two biological parents, 0 = Others
	The educational levels of the participants’ parents and information on the adolescent´s family type were obtained from the questionnaire data collected when the participants were 16 years old.

Tertiary education was used as the primary proxy for human capital, given its strong and consistent association with long-term earnings and productivity [36]. This variable was selected a priori due to its well-established theoretical and empirical relevance in life-course and economic research [37, 38]. Educational attainment remains the most widely accepted indicator of human capital, as it reflects an individual’s accumulated skills and economic potential [39, 40]. In our previous study using the same cohort [41], we showed that adolescent symptoms of ADHD, ODD, and particularly their co-occurrence, were associated with lower educational attainment in adulthood. While tertiary education served as our main proxy, additional indicators of human capital—work experience and occupational status—were also included to complement the analysis (Table 1). Occupational status was classified using a white-collar framework: upper white-collar included managerial, professional, and higher-level administrative roles; lower white-collar referred to clerical and lower-level administrative positions. All other categories including manual workers, students, pensioners, and others were coded as 0.

Interpersonal trust was used as the primary proxy for social capital, based on its strong conceptual and empirical grounding in the literature. Trust plays a foundational role in the development of social networks that enable access to information and opportunities [15, 42, 43]. Among various indicators, trust has consistently emerged to be a robust and least prone to endogeneity concerns, particularly in relation to economic outcomes [34, 35, 44]. While variables such as marital status [45] and parenthood [46] have been linked to aspects of social capital, the empirical support for trust as a core indicator of social capital is stronger [34, 35]. Marital status and parenthood by age 30 were included as additional indicators of social capital in this study (Table 1).

Health capital was proxied by the presence of psychiatric disorders other than ADHD or ODD, given their consistent association with reduced earnings, particularly when onset occurs during adolescence [47–49]. Psychiatric comorbidity with ADHD is more the rule than the exception, and previous studies suggest that disorders such as anxiety, depression, and bipolar disorder alongside ADHD tend to further worsen occupational outcomes [50, 51]. This supports the role of co-occurring psychiatric conditions as a meaningful indicator of compromised health capital in early adulthood. Diagnoses were obtained from the Care Register for Health Care maintained by the Finnish Institute for Health and Welfare, covering specialized inpatient care (through 2016) and outpatient care (1998–2016), and primary care outpatient treatments (2011–2016). If participants had only ADHD or ODD in the health notifications, they were not considered in connection with this variable: 314.0B and 314.1 A were used as diagnostic criteria for ADHD and 313.8 A for ODD in ICD9, while F90.0 was used as a diagnostic criterion for ADHD and F91.3 for ODD in ICD10. Participants were classified as having a psychiatric disorder if at least one diagnosis other than ADHD or ODD was recorded (Table 1).

Self-reported health, while often used as a measure of perceived physical well-being and shown to correlate with objective health indicators [52, 53], was not used as a primary health capital measure in this study due to its subjectivity and early measurement at age 16.

### Covariates

Numerous factors influencing occupational outcomes and income have been identified in prior research, many of which are also associated with ADHD and ODD [7, 54, 55]. To better isolate the associations between adolescent ADHD and ODD symptoms and adult income, we adjusted the multiple mediation model for several covariates.

Parental education and family type during adolescence were included to account for early socioeconomic background (Table 1). Parental education, reported via questionnaire when participants were 16 years old, was coded as 1 for tertiary education (i.e., a university or university of applied sciences degree) and 0 for all other educational levels. Family structure was based on adolescent questionnaire data and coded as 1 if the participant lived with both biological parents, and 0 for all other family arrangements.

We also include the remaining variables used in the simple mediation analyses as covariates in the full model, as they are associated with both income and the selected mediator variables (see Supplementary Tables S1 and S2 for correlations between variables). Given that the correlation coefficients between the mediators and covariates range from 0.596 to − 0.151 for males and from 0.410 to − 0.169 for females, there is no indication of problematic multicollinearity that would substantially bias model estimation or prediction [56, 57]. Additional covariates in the mediation analyses included work experience, white-collar status, marital status, parenthood, and self-rated health (Table 1).

### Statistical methods

We used mediation analyses to explore how ADHD and ODD symptoms in adolescence affect adult incomes. The direct effect is shown by the income difference between individuals with and without these symptoms, while the indirect effect is concerned with how these symptoms influence life choices, such as education, career, social interactions, and health, which in turn affect incomes.

**Fig. 1:**
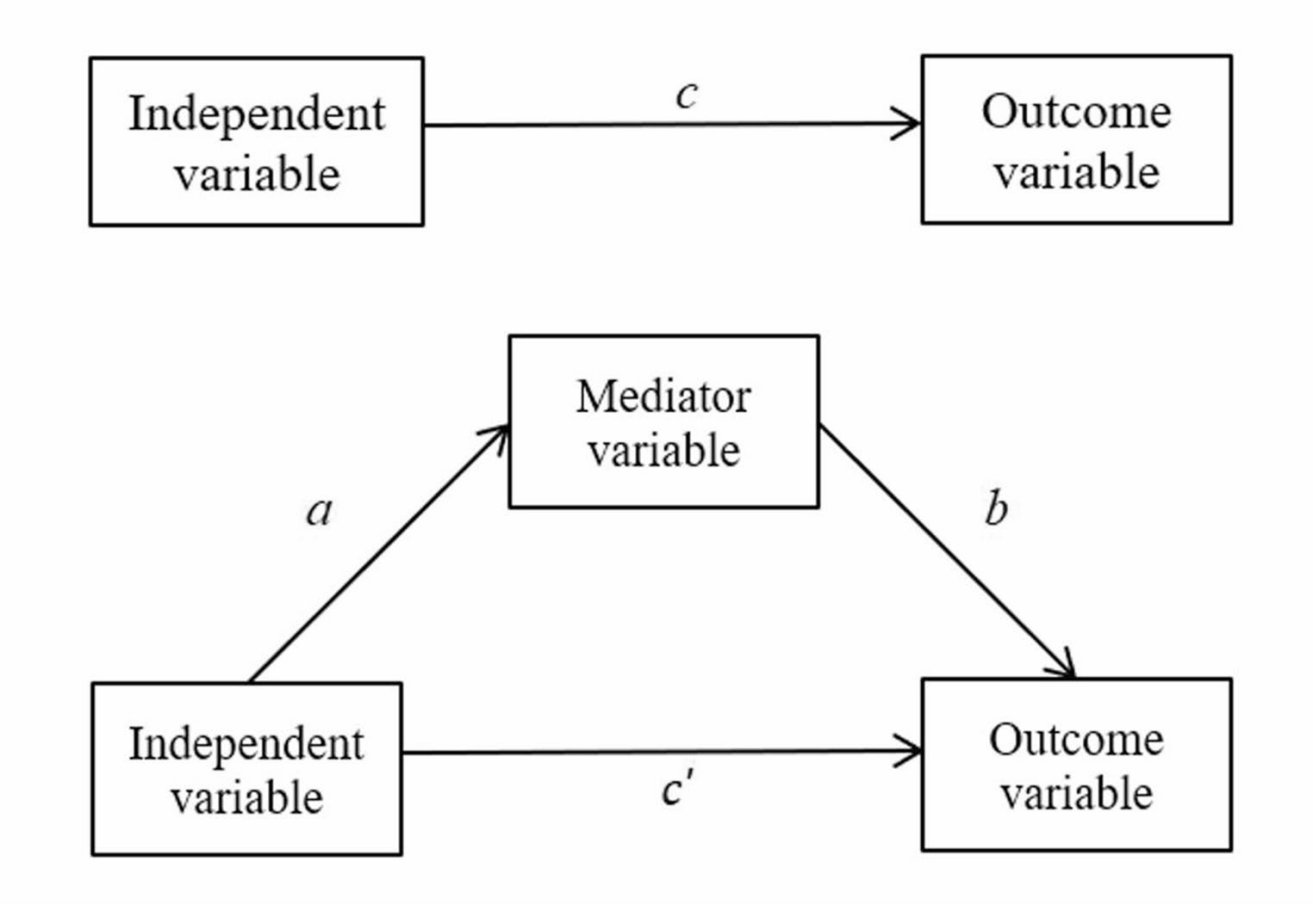
The direct effect of an independent variable on an outcome variable and the effect of mediation

At a general level, mediation processes can be expressed in terms of mediator variables operating between an independent variable and an outcome variable, i.e. a mediator variable is thought of as transmitting the effect of the independent variable to the outcome variable [58]. The total effect of the independent variable on the outcome variable, c, can thus be partitioned into a combination of a direct effect, c’, and an indirect effect transmitted through the mediator variable, a*b (Fig. 1). In other words, the relationship between an independent variable and an outcome variable is decomposed into a direct link and an indirect link, i.e. c = c’ + a*b. Within a regression framework, the population parameters representing the direct and indirect effects are estimated with a set of two individual regression models [58].

We conducted the mediation analyses as recommended by Iacobucci (2012) [59], using the “products of coefficients” approach [60]. First, we computed the mediated effects of ADHD and/or ODD symptoms for each mediator individually, and then we expanded the simple mediation tests by performing a multiple mediation analysis using one proxy variable for each form of capital: educational attainment for human capital, interpersonal trust for social capital, and the presence of psychiatric disorders other than ADHD or ODD for health capital. This approach was chosen to reduce model complexity and minimize potential multicollinearity among variables.

**Fig. 2:**
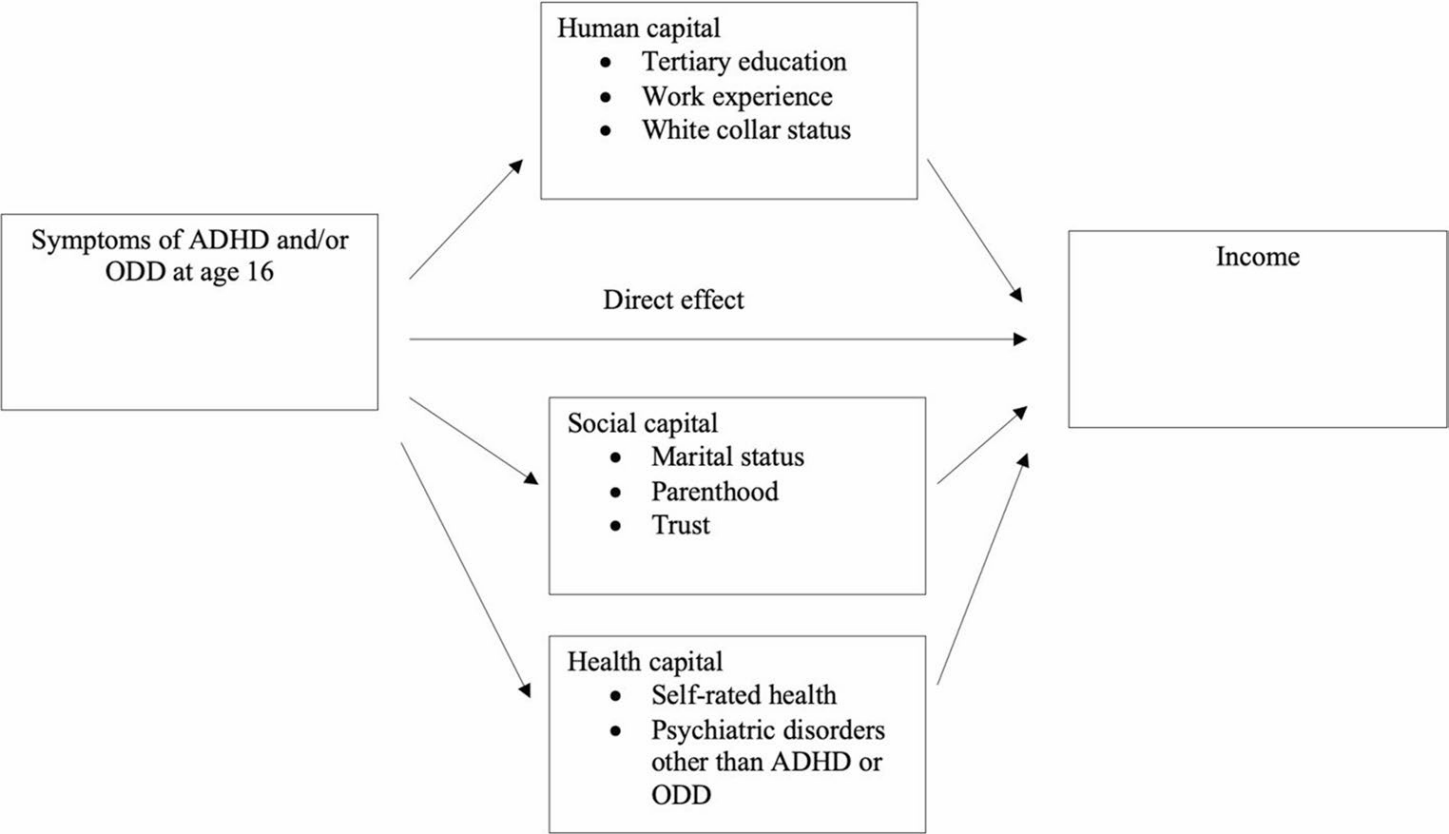
The proposed relationship between ADHD and/or ODD symptoms in adolescence and income in adulthood

All the analyses were conducted separately by sex, and the indirect effects of adolescent ADHD and ODD symptoms on adult incomes were presumed to be mediated through constructs that we refer to as human, social, and health capital (Fig. 2).

The independence of the means in the contingency tables was assessed using the Pearson χ² test for categorical variables and an analysis of variance (ANOVA), with the Welch test applied where necessary, for continuous variables. Post hoc tests were performed using the Tukey test and the Games-Howell test, as appropriate. Income was modelled in natural logarithmic form in the mediation analyses, thus allowing the results to be expressed as percentage changes by multiplying the estimated coefficients by 100. All the statistical analyses were performed using Stata 16.0 (StataCorp LLC), and all the tests were two-tailed, with *p*-values below 0.05 considered statistically significant.

We corrected for multiple testing across 8 simple mediation models using the Benjamini-Hochberg method [61, 62]. A Bonferroni correction [63, 64] was applied to account for six statistical tests per group in the multiple mediation analysis: three specific indirect effects (one per mediator), the total indirect effect, the direct effect, and the total effect. Adjusted significance thresholds were set at 0.00833 (i.e., 0.05/6), 0.00167 (i.e., 0.01/6), and 0.000167 (i.e., 0.001/6).

## Results

Descriptive statistics for the ADHD, ODD, and ADHD + ODD symptom groups, categorized by sex, are presented in Table 2. Males made up 66.3% of the participants with ADHD symptoms, whereas slightly more females than males (55.1%) were affected by ODD symptoms.

**Table 2:** Population characteristics

	Controls (*N* = 2,672)	ADHD (*N* = 236)	ODD (*N* = 61)	ADHD + ODD (*N* = 111)	Anova/Welch/$\:{\chi\:}^{2}$(df), *p*-value	Post hoc test^a^
Males (*N* = 3,080)						
Avg. income in €’s (SD)	30,060 (16,700)	26,950 (15,290)	26,120 (18,660)	24,890 (16,300)	6.54 (3), *p* < 0.001	C > A/AO (T)
Median income in €´s	30,120	25,890	26,390	25,270	–	–
*Human capital*						
Tertiary education	1,128 (42.3)	33 (14.3)	20 (32.8)	11 (10.1)	110.67 (3), *p* < 0.001	C > A/O/AO (G)
Avg. work experience (SD)	4.5 (2.0)	4.1 (2.2)	3.8 (2.3)	3.8 (2.3)	7.22 (3), *p* < 0.001	C > A/AO (G)
White collar	1,118 (42.3)	53 (22.6)	15 (24.6)	23 (20.7)	59.13 (3), *p* < 0.001	C > A/O/AO (G)
*Social capital*						
Marital status	790 (29.6)	56 (23.7)	20 (32.8)	25 (22.5)	6.29 (3), *p* = 0.098	
Parenthood	1,033 (38.7)	104 (44.1)	21 (34.4)	49 (44.1)	4.40 (3), *p* = 0.222	
Trust	2,191 (89.5)	179 (88.2)	46 (85.2)	72 (80.0)	7.79 (3), *p* = 0.051	
*Health capital*						
Self-rated health	321 (13.1)	39 (18.8)	14 (25.9)	25 (27.2)	25.03 (3), *p* < 0.001	C < AO (G)
Psych. disorders^b^	416 (15.6)	61 (25.8)	17 (27.9)	47 (42.3)	70.07 (3), *p* < 0.001	C < A/AO, A < AO (G)
*Covariates*						
Parents’ education, high	537 (20.6)	32 (14.0)	13 (21.7)	11 (10.6)	14.13 (6), *p* = 0.028	C > AO (T)
Family type during adolescence	2,056 (79.9)	144 (64.6)	41 (70.7)	101 (57.4)	55.17 (3), *p* < 0.001	C > A/AO (G)
Females (*N* = 3,083)						
Avg. income in €’s (SD)	22,650 (13,120)	19,900 (11,380)	20,650 (11,780)	18,760 (10,930)	4.52 (3), *p* = 0.004	C > AO (T)
Median income in €`s	22,570	20,640	19,370	19,560	–	–
*Human capital*						
Tertiary education	1,700 (60.7)	36 (30.0)	36 (48.0)	18 (21.2)	97.26 (3), *p* < 0.001	C > A/O/AO (G)
Avg. work experience (SD)	4.5 (1.8)	4.0 (2.0)	4.3 (1.9)	3.7 (2.2)	6.03 (3), *p* < 0.001	C > A/AO (G)
White collar	1,730 (63.2)	58 (50.0)	44 (61.1)	40 (47.6)	16.22 (3), *p* = 0.001	C > A/AO (G)
*Social capital*						
Marital status	1,110 (39.6)	46 (38.3)	22 (29.3)	22 (25.9)	9.54 (3), *p* = 0.023	C > AO (G)
Parenthood	1,536 (54.8)	76 (63.3)	45 (60.0)	50 (58.8)	4.51 (3), *p* = 0.211	
Trust	2,602 (96.9)	105(97.2)	68 (97.1)	73 (92.4)	5.24 (3), *p* = 0.155	
*Health capital*						
Self-rated health	411 (15.3)	34 (31.5)	22 (31.4)	27 (34.2)	48.98 (3), *p* < 0.001	C < A/O/AO (G)
Psych. disorders^b^	616 (22.0)	48 (40.0)	28 (37.3)	38 (44.7)	50.72 (3), *p* < 0.001	C < A/O/AO (G)
*Covariates*						
Parents’ education, high	525 (19.1)	12 (10.1)	14 (19.2)	8 (9.9)	13.38 (6), *p* = 0.037	C > A/AO (T)
Family type during adolescence	2,096 (77.5)	85 (74.6)	46 (63.0)	59 (72.8)	9.49 (3), *p* = 0.023	C > O (G)

^a^Pairwise crosstabulations between the groups; only significant differences (*p* < 0.05) are noted. bOther than ADHD or ODD. *A* ADHD, *O* ODD, *AO* ADHD + ODD, *C* controls. (*T* Tukey, *G* Games-Howell). *ADHD* Attention-Deficit/Hyperactivity Disorder, *ODD* Oppositional Defiant Disorder. Biological sex was recorded at birth and obtained from cohort registry data.

All the symptomatic groups had lower annual incomes than the control group, but this association was statistically significant in the Post Hoc comparisons only for the male ADHD and ADHD + ODD groups and the female ADHD + ODD group. Because the median incomes closely matched the means across groups, the observed income differences are unlikely to be substantially influenced by extreme values. In terms of human capital, all the male and female symptomatic groups achieved lower rates of tertiary education than the control group, while the average duration of work experience was lower for both males and females in the ADHD and ADHD + ODD groups than among the controls. Regarding socioeconomic status, all the male symptomatic groups were less likely to have white-collar jobs than the control group, a trend that was also observed among the females in the ADHD and ADHD + ODD groups. Concerning social capital, the females in the ADHD + ODD group were less likely to be married than those in the control group, but otherwise the social capital variables did not show any clear trends.

In terms of health capital, the males in the ADHD + ODD group reported poorer self-rated health than the controls, and the male ADHD and ADHD + ODD groups both had higher rates of other psychiatric disorders than did the control group. Among the females, all the symptomatic groups reported poorer self-rated health and had more psychiatric comorbidities than the controls.

The results of the simple mediation analyses i.e. the mediated effects of ADHD, ODD, and ADHD + ODD symptoms in adolescence calculated for each mediator individually, by sex, are presented in Table 3.

**Table 3:** Results of the simple mediation analyses

	ADHD		ODD		ADHD + ODD	
Mediator	Ind. eff.	*p*-value	Ind. eff.	*p*-value	Ind. eff.	*p*-value
	Males					
*Human capital*						
Tertiary education	−0.226***	< 0.001	−0.081	0.193	−0.288**	0.004
Work experience	−0.044*	0.011	−0.043	0.286	−0.040	0.211
White collar	−0.198***	< 0.001	−0.144	0.051	−0.202***	< 0.001
*Social capital*						
Marital status	−0.042	0.504	0.051	0.704	−0.053	0.999
Parenthood	0.029	0.102	−0.003	0.999	0.035	0.456
Trust	−0.003	0.999	−0.018	0.999	−0.060	0.156
*Health capital*						
Self-rated health	−0.047	0.160	−0.078	0.088	−0.092*	0.019
Psych. disorders^a^	−0.171***	< 0.001	−0.096	0.999	−0.290***	< 0.001
	Females					
*Human capital*						
Tertiary education	−0.255***	< 0.001	−0.109	0.121	−0.361***	< 0.001
Work experience	−0.035	0.076	−0.023	0.538	−0.036	0.150
White collar	−0.192**	0.002	−0.009	0.999	−0.125	0.224
*Social capital*						
Marital status	0.0004	0.999	0.015	0.747	0.028	0.341
Parenthood	−0.090	0.152	−0.080	0.270	−0.044	0.999
Trust	−0.0003	0.999	0.001	0.999	−0.001	0.999
*Health capital*						
Self-rated health	−0.085**	0.008	−0.083*	0.019	−0.101**	0.005
Psych. disorders^a^	−0.092**	0.005	−0.081*	0.042	−0.087*	0.031

^a^Other than ADHD or ODD. Notes: Ind. eff. is the indirect effect of ADHD/ODD/ADHD + ODD group on annual incomes in 2016 operating through the mediator relative to the control group. Effects are presented in terms of relative changes in annual income in 2016 for a unit change in the regressors. The indirect effect of ADHD and ODD symptoms was calculated for each mediator separately. Work experience is continuous variable with units in years, while the other mediators are categorical. *, * *, and *** represent statistical significance at the 5%, 1%, and 0.1% levels, respectively. p-values are calculated from robust standard errors and adjusted for multiple comparisons using the Benjamini–Hochberg procedure. *ADHD* Attention-Deficit/Hyperactivity Disorder (ADHD), *ODD* Oppositional Defiant Disorder.

Human capital variables education, work experience, and white-collar status mediated the association between adolescent ADHD symptoms and annual incomes in adulthood for males relative to the control group, while education and white-collar status did so for females (Table 3). Education and white-collar status also mediated the association between adolescent ADHD + ODD symptoms and annual income in adulthood for males, while education alone did so for females.

Certain health capital variables also mediated the association between adolescent ADHD symptoms and adulthood incomes, namely self-rated health and the presence of psychiatric disorders other than ADHD or ODD in the females. This mediation effect was also observed in the female ODD group and ADHD + ODD group. In the male ADHD group other psychiatric disorders mediated the association with incomes, and both self-rated health and other psychiatric disorders did so in the male ADHD + ODD group.

Social capital variables, including marital status, parenthood, and trust did not mediate the association between any symptomatic groups and annual incomes in adulthood.

The results of the multiple mediation analyses are presented in Table 4, in which for simplicity, a single proxy is used for each type of capital: education for human capital, trust for social capital and the presence of psychiatric disorders other than ADHD or ODD for health capital.

**Table 4:** Results of the multiple mediation analyses

	ADHD		ODD		ADHD + ODD	
	beta	*p*-value	beta	*p*-value	beta	*p*-value
Panel A. Unadjusted Model						
Indirect effects	Males					
Human capital (education)	−0.238***	< 0.0001	−0.079	0.146	−0.315***	< 0.0001
Social capital (trust)	−0.004	0.885	−0.018	0.874	−0.063	0.071
Health capital (psych. disorders^a^)	−0.173***	0.0002	−0.093	0.418	−0.316***	< 0.0001
Total indirect effect	−0.415***	< 0.0001	−0.190	0.306	−0.694***	< 0.0001
Direct effect	−0.059	0.072	0.061	0.365	−0.032	0.500
Total effect	−0.474***	< 0.0001	−0.129	0.516	−0.726***	< 0.0001
Indirect effects	Females					
Human capital (education)	−0.287***	< 0.0001	−0.112	0.088	−0.437***	< 0.0001
Social capital (trust)	0.005	0.978	−0.011	0.979	0.015	0.930
Health capital (psych. disorders^a^)	−0.074*	0.007	−0.065	0.048	−0.070	0.034
Total indirect effect	−0.356	0.053	−0.188	0.659	−0.492	0.011
Direct effect	−0.011	0.778	−0.015	0.757	−0.002	0.964
Total effect	−0.367	0.051	−0.203	0.635	−0.494	0.013
Panel B. Full Model						
Indirect effects	Males					
Human capital (education)	−0.190***	< 0.0001	−0.063	0.164	−0.252**	0.0002
Social capital (trust)	−0.002	0.890	−0.012	0.912	−0.042	0.111
Health capital (psych. disorders^a^)	−0.097***	< 0.0001	−0.052	0.466	−0.177***	0.0001
Total indirect effect	−0.289***	< 0.0001	−0.127	0.382	−0.471***	< 0.0001
Direct effect	−0.020	0.496	0.089	0.130	−0.010	0.830
Total effect	−0.309***	< 0.0001	−0.038	0.807	−0.481***	< 0.0001
Indirect effects	Females					
Human capital (education)	−0.161***	< 0.0001	−0.063	0.095	−0.245***	< 0.0001
Social capital (trust)	0.006	0.970	−0.015	0.971	0.020	0.900
Health capital (psych. disorders^a^)	−0.041	0.030	−0.036	0.080	−0.039	0.062
Total indirect effect	−0.196	0.256	−0.114	0.787	−0.264	0.116
Direct effect	0.035	0.337	0.005	0.910	0.039	0.378
Total effect	−0.161	0.356	−0.109	0.795	−0.225	0.196

^a^Other than ADHD or ODD. Notes: The indirect effects are the effects of ADHD, ODD or ADHD + ODD on annual incomes in 2016 through the mediators, calculated relative to the control group. The total indirect effect is the sum of the three indirect effects, and the total effect is the sum of the total indirect effect and the direct effect. The full model in Panel B includes controls for work experience, white collar status, marital status, parenthood, self-rated health, educational attainments of the participants´ parents, and the family type during adolescence. *, **, and *** represent statistical significance at the 5% (p < 0.00833), 1% (p < 0.00167), and 0.1% (p < 0.000167) levels, respectively, after Bonferroni correction for 6 tests. p-values are calculated from bootstrapped standard errors using 5000 replications. *ADHD* Attention-Deficit/Hyperactivity Disorder (ADHD), *ODD* Oppositional Defiant Disorder.

The unadjusted model results in Panel A of Table 4 indicate that the ADHD and ADHD + ODD symptom groups have a significant indirect negative effect on the incomes of males that is mediated through human capital and health capital. The largest indirect effect is via human capital (education), accounting for over half of the total indirect effect. Among females, ADHD symptoms showed significant indirect effects on income through both human capital and health capital separately. In addition, ADHD + ODD symptoms showed a significant indirect effect via human capital. However, neither the total indirect effect nor the total effect (combining indirect and direct effects) reached statistical significance.

The full multiple mediation model in Panel B of Table 4 shows that the results remain statistically significant even though the magnitude of the effects is smaller than in the unadjusted model due to the additional controls. The effects mediated by both human and health capital remain statistically significant for the males with ADHD or ADHD + ODD, while for the females, the effect mediated by human capital remains statistically significant for both the ADHD and ADHD + ODD groups.

There is no direct effect of ADHD or ADHD + ODD symptoms on incomes for males or females in either Panel A or Panel B, nor is there any direct or indirect effect of ODD symptoms.

In conclusion, there is a mediated effect of ADHD and ADHD + ODD symptoms on incomes for both males and females, this being strongest for the male ADHD + ODD group. The mediated effect of ADHD + ODD symptoms on the average income is −25% through human capital (education) and − 18% through health capital (psychiatric disorders other than ADHD or ODD) in the full model. The total mediated effect for the male ADHD + ODD group was − 47%. By comparison, the mediated effect of ADHD symptoms on average incomes for females is −16% via human capital.

The lack of significant interaction effects in Supplementary Table 3 (Table S3) supports our model framework, indicating that the effect of ADHD and ADHD + ODD symptoms on income is mediated by the human and health capital measures considered here rather than moderated by them.

Social capital did not mediate the association between symptoms of ADHD, ODD, or ADHD + ODD and incomes, as evidenced by the statistically significant coefficients in the interaction analyses (Table S3).

## Discussion

It was found here that ADHD and ADHD + ODD symptoms in adolescence indirectly affect adult incomes through human and health capital, but not through social capital, with stronger effects observed in subjects with both ADHD and ODD symptoms. More specifically, the indirect effects of ADHD + ODD symptoms on average income were − 25% through education and − 18% through co-occurring psychiatric disorders (other than ADHD or ODD). Taken overall, the total indirect effect of adolescent ADHD + ODD symptoms on average adult incomes for males was − 47%. The model was adjusted for various factors, including work experience, white-collar status, marital status, parenthood, self-rated health, educational attainments of the participants’ parents, and family type during adolescence. Notably, there was no direct effect of ADHD and/or ODD symptoms in adolescence on adult incomes.

Previous studies have suggested that education partially mediates the link between ADHD and occupational outcomes, though the findings are mixed. A longitudinal, register-based study performed in Sweden [54], for instance, demonstrated that while educational attainment partially explained the connection between ADHD and occupational outcomes, disparities between individuals with and without ADHD persisted over time regardless of the level of education. One genetically sensitive study found that education played a minor role in occupational outcomes among siblings with differing ADHD symptoms [65], while another found that it significantly mediated the relationship between a polygenic ADHD score and income [66]. Our findings are in line with the above, suggesting a significant indirect association via education between adolescent ADHD symptoms and adult incomes.

Measuring health capital via self-rated health and the presence of psychiatric disorders other than ADHD or ODD, we found in our multiple mediation analyses that co-occurring psychiatric disorders mediated the relationship between adolescent ADHD symptoms and adult incomes for both males and females. This is consistent with previous suggestions that psychiatric comorbidities in addition to ADHD itself play a substantial role in occupational outcomes. One European study, for instance, reported a link between psychiatric comorbidities and higher unemployment rates among adults with ADHD [67]. In addition, research has shown that employment rates are comparable between individuals with ADHD and a clinical control group with other psychiatric conditions but without an ADHD diagnosis [68]. It is significant, however, that the Swedish study excluded ADHD-related psychiatric comorbidities, such as depression, from their analyses, citing concerns about potential moderating effects. They argued that these comorbidities could be influenced by both ADHD and occupational outcomes, such as income, thereby complicating the relationship between ADHD and career success [54]. In contrast, our study included psychiatric comorbidities but found no evidence of a moderating effect.

ADHD and/or ODD symptoms were not associated with social capital variables such as marital status, parenthood, or trust when compared to controls in the present instance, although there was a statistical trend (*p* = 0.051) in the one-way ANOVA, suggesting that trust was more prevalent among the controls than in the symptomatic groups. As a result, social capital (trust) did not mediate the relationship between these symptomatic groups and adult incomes. These findings are somewhat unexpected in the light of previous research. For instance, it has been reported from Norway that the relationship between ADHD symptoms and occupational outcomes was entirely mediated by social and role-emotional functioning [69].

While both human and health capital mediated the association between adolescent ADHD symptoms and adult incomes in the present population, social capital (trust) did not have the same effect. Finland’s high standard of living and powerful social safety nets for single-parent and low-income families may have contributed to this outcome. Interpersonal relationships are crucial to the psychosocial development of adolescents, and a lack of friendships can result in poor social skills and eventual withdrawal from the community´s social networks. Although low levels of trust are linked to loneliness [70], some adolescents may compensate for their social deficits by channelling their efforts into academic and occupational achievements in order to maintain a positive self-image. Alternatively, traits such as risk-taking and entrepreneurial tendencies could reduce reliance on traditional social interactions in one´s career pathways.

The fact that we found no direct or indirect effect of ODD symptoms on incomes for either males or females may be related to the stronger impact of disruptive behaviour on academic achievement during the earlier stages of life, such as adolescence and early adulthood, i.e. the effects of ODD symptoms may diminish as individuals mature and age. Previous research into the association between ODD symptoms and income has yielded inconsistent results. The Columbia County Longitudinal Study, for example, showed that aggressive behaviour in childhood or adolescence negatively affected educational status in early adulthood, which in turn predicted a poorer occupational status in middle adulthood [8]. Likewise, the Jyväskylä Longitudinal Study of Personality and Social Development reported that teacher-rated aggression at age eight predicted school maladjustment at age 14, which in turn was both directly and indirectly related to long-term unemployment (via problem drinking and a lack of occupational alternatives at age 27) [71].

### Strengths and limitations

The principal strength of this study lies in its large, population-based sample and longitudinal prospective design, which together provide robust insights through comprehensive statistical analyses. The NFBC1986 cohort has maintained high participation rates throughout its follow-ups, with 76% of the parents and 80% of the included in the 16-year follow-up. Also, the sample data are linked to accurate Finnish national registers, providing reliable information on participants’ incomes and other variables. This linkage will enhance the reliability of the data and minimize attrition bias. Another notable strength of the study is the use of symptom-based groupings rather than formal diagnoses, enabling the identification of individuals with high symptom levels in a population-based setting. Although we used a 95th percentile cutoff, this approach retains a dimensional perspective and captures individuals who may not meet diagnostic criteria but still face functional challenges.

Some limitations should be acknowledged. Our approach relies on the assumption that ADHD/ODD symptoms are exogenous to both income and the mediators, conditional on observed covariates. Symptoms were measured in adolescence and income was assessed in adulthood, thereby establishing a clear temporal ordering. This timing supports the plausibility of treating the symptoms as exogenous to income, although the possibility of unmeasured confounding remains. Unmeasured confounding may bias estimates if factors such as personality traits or time preferences affect both mediators and income [72, 73]. Some mediators were measured at age 16, potentially overlapping symptom assessment. This raises temporal concerns, especially given the theoretical role of mediators as downstream mechanisms. Likewise, income was measured at age 30, providing only a snapshot of early adulthood earnings, which may not capture long-term economic trajectories. In addition, excluding participants with zero income—necessary for log-transforming the income variable—may slightly limit generalizability to those fully detached from the labor market or facing severe economic hardship. However, this improves the robustness and interpretability of the estimates and likely has minimal impact, as these cases represent a small share of the sample. Furthermore, ADHD and ODD symptoms were assessed at age 16, a developmental stage at which puberty-related changes may amplify the expression of symptoms, potentially introducing an element of bias [74].

Group definitions were based on a pooled (non-sex-specific) 95th percentile cutoff, which may underrepresent symptom severity in females due to known sex differences in symptom distribution and expression. This may affect cross-sex comparability and should be interpreted accordingly. Fewer findings were observed for the ODD-only group, which may reflect true attenuation of effects over time as noted in the discussion. However, the small group size likely limited statistical power to detect effects and must also be considered when interpreting null findings. Furthermore, the study is drawn from two provinces in Finland and the cohort is ethnically and socioeconomically homogenous. While this minimizes cultural variability, it limits generalizability to more diverse populations and contexts.

Using a single proxy for each capital domain may oversimplify complex constructs. Our alternative indicators of human capital—work experience and occupational status—were less appropriate given the relatively young age of participants (30 years), which limits accumulated work experience. Moreover, occupational status is strongly shaped by educational attainment, making it less suitable as an independent proxy. Despite this, tertiary education remains a widely accepted indicator of human capital, reflecting skill acquisition and long-term economic potential. Interpersonal trust—measured as having a close friend at age 16—was used as the primary proxy for social capital. While this measure is conceptually grounded and supported by prior research, it is not exhaustive and exhibited limited variability, with over 80% of participants reporting at least one close friend. Combined with its early measurement, this may reduce its ability to meaningfully differentiate between individuals and limit its relevance for adult social functioning. Alternative indicators, including marital status and parenthood at age 30, were explored in sensitivity analyses. However, these variables may reflect broader structural or socioeconomic factors rather than core dimensions of social capital, and their conceptual fit may be considered outdated in contemporary social research. Nonetheless, within the NFBC1986—where early family formation remains relatively common and socially normative—these indicators may retain some contextual relevance, though this naturally limits generalizability. Health capital was proxied by the presence of psychiatric disorders other than ADHD or ODD, due to their established association with reduced earnings. Although self-reported health was available, it was not used as a primary proxy due to its subjectivity and early measurement at age 16. While proxies were selected a priori based on theoretical and empirical relevance, future research should consider more sensitive, multidimensional, and temporally appropriate measures to better capture the roles of human, social, and health capital.

## Conclusions

It was found here that both ADHD and combined ADHD + ODD symptoms experienced in adolescence could indirectly affect adult incomes, the impact being mediated through human capital (education) and health capital (co-occurring psychiatric disorders). Social capital (trust) did not mediate this association, and contrary to previous research, we observed no direct effect of adolescent ADHD or ODD symptoms on adult incomes. Our mediation analyses highlighted the fact that it is not the ADHD symptoms themselves that reduce incomes, but rather their influence through educational attainments and health. These findings have significant socio-economic implications. Enhanced student counselling and early screening for psychiatric symptoms could help narrow the income gap between individuals with ADHD or ODD symptoms and the general population. Future research should focus on gaining a deeper understanding of how ADHD and ODD symptoms affect actual work performance and use more nuanced, multidimensional, and developmentally aligned measures of human, social, and health capital constructs.

## Supplementary Information

Below is the link to the electronic supplementary material.

ESM 1 (DOCX 24.9 KB)

## Data Availability

No datasets were generated or analysed during the current study.
