# Supplementary material for: Indirect associations between adolescent ADHD and/or oppositional defiant disorder symptoms and adult incomes: the mediating roles of education and co-occurring psychiatric disorders
Source: Eur Child Adolesc Psychiatry. 2025 Sep 2;35(2):449–62. doi: 10.1007/s00787-025-02842-2 (PMC12956917; doi:10.1007/s00787-025-02842-2)
Supplement: Supplementary file 1 — (DOCX 24.9 KB) [file 787_2025_2842_MOESM1_ESM.docx]

**Supplementary material**

**Table S1. Correlations between variables for the subsample of males.**

|  | Income | Tertiary education | Avg. work experience | White collar | Marital status | Parenthood | Trust | Self-rated health | Psych. disorders | Parents’ education |
| --- | --- | --- | --- | --- | --- | --- | --- | --- | --- | --- |
| Tertiary education | **0.232** |  |  |  |  |  |  |  |  |  |
| Avg. work experience | **0.392** | **-0.049** |  |  |  |  |  |  |  |  |
| White collar | **0.271** | **0.596** | **0.136** |  |  |  |  |  |  |  |
| Marital status | **0.184** | **0.070** | **0.158** | **0.080** |  |  |  |  |  |  |
| Parenthood | **0.108** | **-0.134** | **0.138** | **-0.077** | **0.491** |  |  |  |  |  |
| Trust | **0.060** | -0.005 | 0.021 | 0.029 | 0.028 | **0.056** |  |  |  |  |
| Self-rated health | **-0.077** | **-0.092** | -0.023 | **-0.091** | **-0.047** | -0.038 | **-0.082** |  |  |  |
| Psych. disorders | **-0.192** | **-0.117** | **-0.151** | **-0.088** | **-0.084** | 0.012 | -0.023 | **0.049** |  |  |
| Parents’ education | **0.054** | **0.151** | -0.031 | **0.099** | 0.027 | **-0.064** | -0.037 | 0.001 | -0.008 |  |
| Family type during adolescence | **0.078** | **0.098** | 0.040 | **0.052** | **0.094** | 0.020 | 0.001 | -0.037 | **-0.099** | 0.016 |

Notes: The table shows Spearman correlation coefficients. Coefficients in bold are significant at the 5% level.

**Table S2. Correlations between variables for the subsample of females.**

|  | Income | Tertiary education | Avg. work experience | White collar | Marital status | Parenthood | Trust | Self-rated health | Psych. disorders | Parents’ education |
| --- | --- | --- | --- | --- | --- | --- | --- | --- | --- | --- |
| Tertiary education | **0.323** |  |  |  |  |  |  |  |  |  |
| Avg. work experience | **0.338** | **0.081** |  |  |  |  |  |  |  |  |
| White collar | **0.382** | **0.332** | **0.364** |  |  |  |  |  |  |  |
| Marital status | **-0.043** | 0.038 | **0.047** | 0.020 |  |  |  |  |  |  |
| Parenthood | **-0.282** | **-0.169** | -0.019 | **-0.096** | **0.410** |  |  |  |  |  |
| Trust | **0.014** | 0.024 | **0.049** | 0.027 | 0.026 | 0.030 |  |  |  |  |
| Self-rated health | **-0.102** | **-0.108** | -0.093 | **-0.070** | **-0.064** | -0.012 | -0.026 |  |  |  |
| Psych. disorders | **-0.147** | **-0.114** | **-0.145** | **-0.130** | **-0.056** | -0.018 | **-0.049** | **0.119** |  |  |
| Parents’ education | **0.069** | **0.127** | -0.020 | **0.048** | 0.018 | **-0.073** | 0.003 | -0.026 | -0.013 |  |
| Family type during adolescence | **0.063** | **0.126** | **0.069** | **0.094** | **0.100** | 0.024 | -0.016 | **-0.056** | **-0.102** | -0.002 |

Notes: The table shows Spearman correlation coefficients. Coefficients in bold are significant at the 5% level.

**Table S3. Interaction of ADHD, ODD or ADHD + ODD symptoms with human, social, and health capital variables.**

|  | Males |  |  |  |  |  |
| --- | --- | --- | --- | --- | --- | --- |
|  | ADHD |  | ODD |  | ADHD + ODD |  |
|  | Coeff. | *p*-value | Coeff. | *p*-value | Coeff. | *p*-value |
| *Human capital* |  |  |  |  |  |  |
| Tertiary education | -0.034 | 0.727 | 0.151 | 0.318 | -0.032 | 0.855 |
| Work exp. (years) | 0.024 | 0.118 | 0.023 | 0.530 | 0.017 | 0.446 |
| White collar | 0.058 | 0.436 | 0.138 | 0.364 | -0.069 | 0.511 |
| *Social capital* |  |  |  |  |  |  |
| Marital status | -0.044 | 0.513 | 0.334* | 0.017 | 0.047 | 0.712 |
| Parenthood | -0.052 | 0.420 | 0.100 | 0.488 | -0.067 | 0.471 |
| Trust | 0.219 | 0.074 | -0.213* | 0.028 | 0.328* | 0.014 |
| *Health capital* |  |  |  |  |  |  |
| Self-rated health | 0.073 | 0.455 | -0.047 | 0.700 | 0.074 | 0.580 |
| Psych. disorders^a^ | -0.069 | 0.407 | -0.342* | 0.028 | -0.060 | 0.563 |
|  | Females |  |  |  |  |  |
|  | ADHD |  | ODD |  | ADHD + ODD |  |
|  | Coeff. | *p*-value | Coeff. | *p*-value | Coeff. | *p*-value |
| *Human capital* |  |  |  |  |  |  |
| Tertiary education | 0.078 | 0.353 | 0.155 | 0.128 | 0.074 | 0.462 |
| Work exp. (years) | 0.007 | 0.710 | 0.001 | 0.956 | 0.018 | 0.422 |
| White collar | -0.079 | 0.286 | 0.069 | 0.564 | -0.058 | 0.568 |
| *Social capital* | 0.126 | 0.150 | -0.092 | 0.380 | 0.073 | 0.513 |
| Marital status |  |  |  |  |  |  |
| Parenthood | 0.169 | 0.075 | -0.092 | 0.365 | 0.013 | 0.894 |
| Trust | -0.023 | 0.816 | 0.362*** | <0.001 | -0.009 | 0.933 |
| *Health capital* |  |  |  |  |  |  |
| Self-rated health | 0.119 | 0.236 | 0.111 | 0.401 | 0.249* | 0.035 |
| Psych. disorders^a^ | 0.050 | 0.576 | -0.067 | 0.537 | -0.065 | 0.545 |

^a^Other than ADHD or ODD. Note: Coeff. is the coefficient of the interaction term (ADHD / ODD / ADHD + ODD group multiplied by a moderator) from the estimated model having only the group (ADHD / ODD / ADHD + ODD), the moderator, and the interaction term as explanatory variables. We ran separate regressions for each moderator. The control group was the group omitted from the equations. Effects are presented in terms of relative changes in annual income in 2016 for a unit change in the regressors. Work experience is a continuous variable with units in years, and the other regressors are categorical. *, * *, and *** represent statistical significance at the 5%, 1%, and 0.1% levels, respectively. *p*-values are calculated from robust standard errors. ADHD = Attention-Deficit/Hyperactivity Disorder, ODD = Oppositional Defiant Disorder.
